# Advancements in carbon nanotube-polymer composites: Enhancing properties and applications through advanced manufacturing techniques

**DOI:** 10.1016/j.heliyon.2024.e36490

**Published:** 2024-08-16

**Authors:** Ermias Wubete Fenta, Berihun Abebaw Mebratie

**Affiliations:** Bahir Dar Institute of Technology, Bahir Dar University, Bahir Dar, Ethiopia

**Keywords:** Carbon nanotubes (CNTs), Polymer composites, Filament extrusion, Additive manufacturing (AM)

## Abstract

Carbon nanotube (CNT)-polymer composites exhibit significant advancements in mechanical, electrical, and thermal properties, enabling numerous promising applications. This review delves into recent research on manufacturing methods, filament extrusion, additive manufacturing (AM), properties, and applications of CNT polymer composites. Factors like processing conditions, polymer types, and CNT concentrations determine the ultimate properties of the composite material. The dispersion of CNT within various manufacturing techniques, such as melt mixing, solution mixing, and in-situ polymerization, significantly impacts the properties of the composite material. These composite materials are extensively used in AM, particularly in 3D printing, where filament blends are extruded and printed to custom-shaped objects. The finding underscores the effect of CNT content on the properties of CNT-polymer composite material in different applications. However, gaps remain in optimizing manufacturing processes and AM techniques, essential for tailoring these composites to specific application needs. Future research should focus on developing cost-effective and scalable manufacturing methods to unlock the full potential of CNT-polymer composites in various industries.

## Graphical abstract

**Figure:**
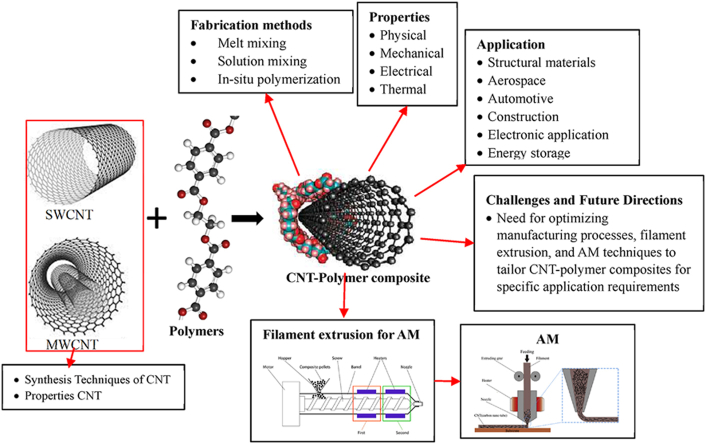

## 1 Introduction

In recent years, there has been a notable trend in the research community towards incorporating carbon nanotubes (CNTs) into polymers to form composite materials. The exceptional qualities of CNTs, such as their impressive mechanical, electrical, and thermal properties, make them highly suitable for reinforcing polymer composites [1,2]. Simultaneously, polymer systems have consistently attracted the attention of manufacturers due to their unique characteristics, including easy processing, lightweight, cost-effectiveness, durability, and often, ductility [[3], [4], [5]]. The unique characteristics of CNTs are considered groundbreaking in the field of materials, positioning them as potential candidates for enhancing polymer matrices due to their remarkable strength and stiffness, low weight, high flexibility, diameter-dependent specific surface area, high aspect ratio, improved thermal and electrical conductivity, as well as moderate electrostatic discharge properties [1,[6], [7], [8], [9]]. The use of polymers is attractive due to their cost-effectiveness, reproducibility, resistance to corrosion, and ease of manufacturing. Various thermoplastic and thermosetting polymers have been utilized as matrices in these efforts. Incorporating CNT into these polymer matrices significantly enhances the resulting composites' conductivity, strength, elasticity, toughness, and durability. This strategy opens up a transformative pathway for developing advanced materials with enhanced performance attributes [[10], [11], [12], [13], [14]].

The manufacturing process of CNT polymer composites involves essential steps to blend these materials effectively and achieve the desired characteristics. It is important to note that the specific manufacturing procedures can vary depending on the type of polymer, the intended application of the composite, and the targeted properties. As a reflection of the evolving nature of this field, ongoing research and development efforts are being made to improve and optimize the production processes for CNT polymer composites, which will guarantee that CNT polymer composites exhibit enhanced properties and better performance for a variety of uses. The formation of CNT polymer composite commonly includes a dispersion process, during which CNTs are incorporated into the polymer. The purpose of dispersion is to evenly disperse each CNT across the matrix material such that the applied load is distributed evenly among the population of nanotubes, enabling to generation of advanced material possessing multifunctional characteristics [[15], [16], [17]]. Over the past two decades, the production of composite filaments for additive manufacturing (AM) has garnered considerable attention from researchers. While conventional thermoplastic polymers serve as the matrix material, a diverse array of fillers has been employed to improve printability, enhancing specific material properties, or introducing new ones [18]. The incorporation of CNT fillers is crucial for achieving enhanced mechanical, physical, electrical, and thermal properties in filaments, facilitating the manufacture of functional components for various industrial applications. One notable advantage of AM using CNT polymer composite material is its capability to produce intricate object geometries that are challenging to achieve through traditional subtractive manufacturing methods, primarily due to the difficulties associated with material subtraction from bulk monoliths [[19], [20], [21]]. The advancement of CNT polymer composites holds the potential for creating lightweight structural materials with versatile functionalities applicable in various domains such as electronic components, micro-batteries, circuits, electromagnetic shielding, electromagnetic wave shielding, heat spreaders, and high-strength structures [8,13,22]. CNT polymer composite materials are being widely utilized in several industrial sectors, including energy, infrastructure, sporting goods, aerospace, defense, automotive, and transportation [8,23].

CNT polymer composites have gained significant attention due to their enhanced mechanical strength, electrical conductivity, and thermal properties, making them ideal for various advanced applications [24,25]. However, there are several areas where improvements in manufacturing methods are needed. Manufacturing of CNT polymer composites primarily involves solution blending, melt blending, and in situ polymerization [26]; however, there is a gap in precisely controlling CNT dispersion, optimizing manufacturing processes for CNT-polymer composites, and developing scalable manufacturing processes appropriate for industrial production. Although AM/3D printing uses CNT polymer composites, filament manufacturing for AM requires a regulated and consistent dispersion of CNTs inside the polymer matrix. Extensive research on the best dispersion techniques, efficient ways to align CNTs during filament extrusion, and the impact of extrusion parameters (such as temperature profiles, screw speed, and die design) on filament quality are lacking in the recent literature. Enhancing the impact of printing parameters (such as nozzle size, printing speed, and layer height) is necessary in addition to enhancing filament quality because these factors have a significant impact on the results of AM/3D printing. The manufacturing process, filament production, and AM/3DP of CNT polymer composites can all be significantly improved by recent developments in optimization tools, such as computational modeling, machine learning algorithms, and experimental design techniques, enabling better properties in their application areas. The purpose of this work was to examine these problems in light of recent findings and to highlight advancements and comprehension in the field of CNT polymer composites. With an emphasis on obtaining improved mechanical, electrical, thermal, and physical properties, by addressing associated challenges thorough analysis of the state of the art in the creation of CNT polymer composite materials is provided in this article. The study examines the effects of varying CNT filler weights with various polymer types to create higher-quality CNT polymer composites, CNT polymer composite filaments, and 3D printed items to improve their mechanical, electrical, thermal, and physical properties. Reviewing the advanced applications of CNT polymer composite, which has better qualities than other materials, was the focus of this study. This review helps the readers to optimize the process parameters for the fabrication of CNT polymer composites, intended to replace current materials characterized by high density, low strength, and limited conductivity. It helps to develop CNT polymer composite materials, filaments, and AM processes that can serve as superior alternatives to existing high-density, low-strength, and less conductive materials without compromising performance indices.

## 2 Materials

**Fig. 1:**
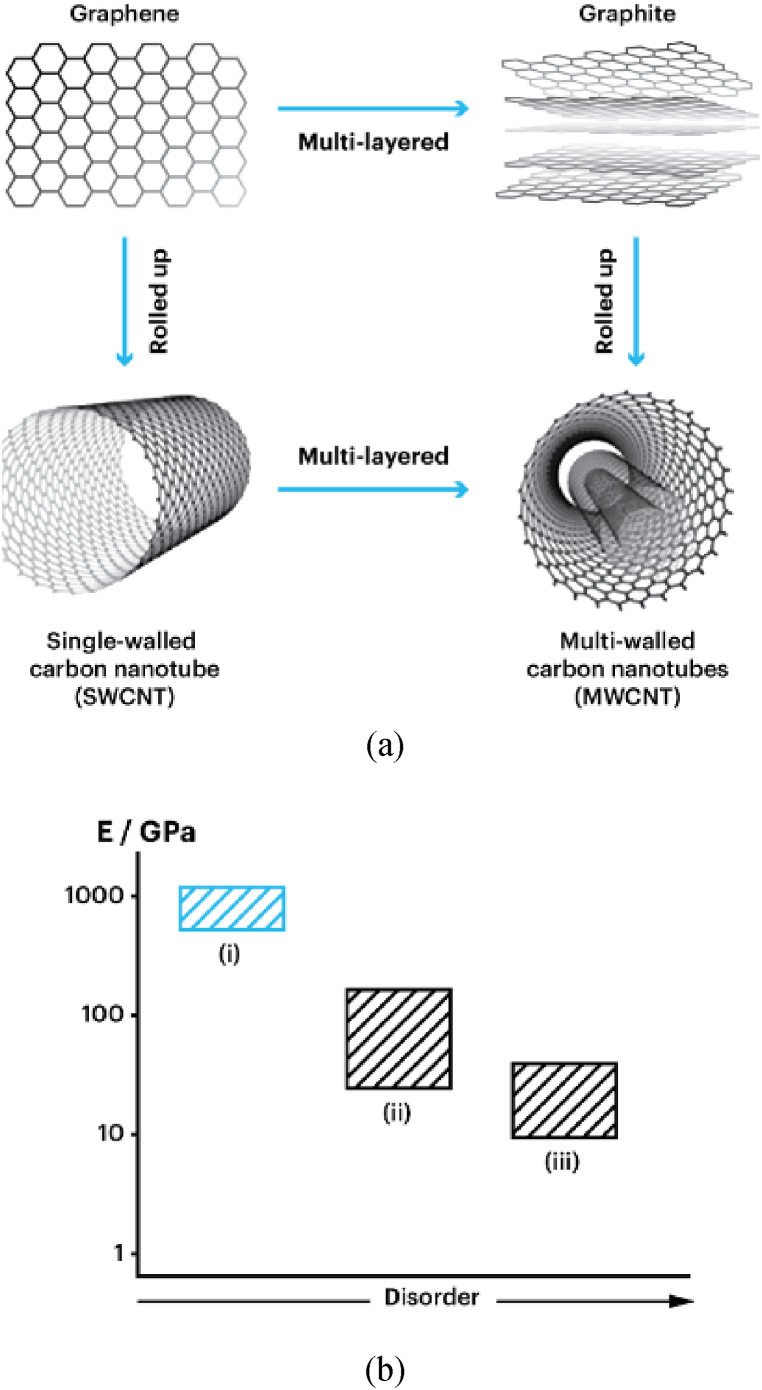
(a) SWCNT and MWCNT structures (b) the dependence of Young's modulus on the CNT structure: (i) SWCNT, (ii) and (iii) MWCNTs [27].

The fabrication of CNT polymer composite material involves utilizing CNT for reinforcement and polymers as the matrix. CNT was chosen as the reinforcement due to its unique physical, mechanical, electrical, and thermal properties, enabling the development of lightweight and electrically conductive composites with enhanced mechanical and thermal characteristics. CNT polymer composite utilizes both single-wall carbon nanotubes (SWCNT) and multi-wall carbon nanotubes (MWCNT). SWCNTs and MWCNTs are derived structures from graphene, a two-dimensional sheet of carbon atoms arranged in a hexagonal lattice, as shown in Fig. 1 (a). The process involves intricate atomic rearrangements where graphene sheets are rolled and stacked to form tubular structures, influencing their properties and potential applications in various fields. Despite their apparent similarities, SWCNTs and MWCNTs exhibit significant differences in properties, as indicated in Fig. 1 (b) and Table 1, due to their structural variances. SWCNTs consist of a single graphene layer forming the nanotube wall, whereas MWCNTs comprise multiple layers of graphene seamlessly rolled into a tubular shape. These basic differences result in differences in their thermal, electrical, and mechanical properties.

**Table 1:** Properties of SWCNT and MWCNT [27,28].

Parameter	SWCNTs	MWCNTs
**Key parameters**		
Typical diameter (nm)	1–2^a^	7–100
Typical length (mm)	Up to 1^b^	Up to 1
Aspect ratio	Up to 10 000	50–4000
**Mechanical properties**		
Elastic modulus (GPa)	1000–3000	300–1000
Tensile strength (GPa)	50–100	10–50
**Electrical properties**		
Electrical conductivity (S/cm)	102–106	103–105
**Thermal properties**		
Thermal conductivity at 300K (W/m.K)	3000–6000	2000–3000
Thermal stability temperature in air (^o^C)	550–650	550–650
Minimum working dosage as an anti-static additive	0.01 %	0.5 %

a Larger diameters are possible but could lead to an increased number of defects.

b Longer lengths are possible but only at laboratory-scale.

**Fig. 2:**
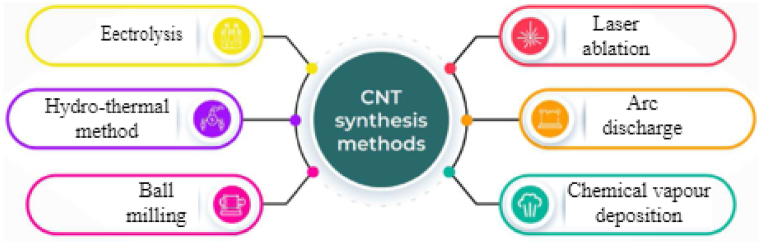
Various synthesis techniques of CNTs [29].

Different techniques have been developed to synthesize CNTs, as depicted in Fig. 2, with specific properties such as size, shape, structure, purity, and surface characteristics. The selection of a synthesis technique depends on the desired type of CNT (single-walled or multi-walled) and its intended use. Researchers continuously refine these methods to produce nanotubes with tailored properties. Three primary techniques dominate CNT production are electric arc discharge, laser ablation, and chemical vapor deposition [[29], [30], [31]].

In CNT-reinforced composites, polymers are the most widely used material due to their cost-effectiveness, reproducibility, resistance to corrosion, and ease of manufacturing processes. Selecting the appropriate polymer matrix during the production of CNT polymer composites is crucial as it impacts the ultimate properties of the composite. Various researchers have utilized thermosetting and thermoplastic polymers as matrix components in CNT polymer composites [[3], [4], [5]]. The selection of a specific polymer matrix depends on the intended application and the desired combination of properties. Polymers like acrylonitrile butadiene styrene (ABS), polyvinyl chloride (PVC), poly methyl methacrylate (PMMA), polyethylene terephthalate (PET), polylactic acid (PLA), polyvinylidene fluoride (PVDF), polyacrylonitrile (PAN), polypropylene (PP), natural rubber (NR), Polyether sulfone (PES), polyurethane, polyimides, and epoxy resins are frequently utilized [23,32]. By adding CNT to these polymers, the resulting material becomes stronger, tougher, more durable, and improved thermal and electrical conductivity. Researchers are continuously experimenting with new combinations of polymers and CNTs to create materials with specific properties for various industries.

The weight percentage of CNTs incorporated into polymer plays a crucial role in determining the physical, mechanical, electrical, and thermal properties of the CNT polymer composite material. From the literature reviewed [33,34], CNT loading ranging from 0 to 60 % by weight has been explored in various studies. However, it is noteworthy that the most commonly employed range for CNT content in CNT polymer composites is between 1 % and 10 % by weight [35]. CNT concentrations of less than 1 % may not significantly impact the composite's properties and concentrations of more than 10 % may cause an increase in the cost of the composite and make it more difficult to achieve uniform CNT dispersion. The optimal CNT loading can vary significantly based on the polymer matrix, application, and desired properties.

## 3 Fabrication methods of CNT polymer composites

In practical applications, different manufacturing techniques have been employed by researchers to fabricate CNT-polymer composites. CNT-polymer composites have excellent properties, but these properties differ depending on fabrication methods, dispersion, orientation of CNTs, CNT length, matrix properties, and chirality [23,36]. The fabrication techniques for CNT polymer composites encompass melt mixing, solution mixing, sonication, resin transfer molding, bucky paper resin infiltration, aligned CNT sheet processing, shear mixing, and in-situ polymerization [26]. Among these, melt mixing, solution mixing, and in-situ polymerization are the most commonly used techniques for producing CNT-polymer composites.

The fabrication process of CNT polymer composites typically involves a step where CNTs are dispersed and incorporated into the polymer matrix. Dispersion helps to uniformly distribute individual CNTs throughout the matrix material, ensuring that applied loads are evenly distributed among the nanotube population [36,37]. Despite CNT having unique properties, faces challenges related to the dispersion of agglomerated CNT during processing, leading to poor interfacial interaction between CNTs and the polymers. In addressing these challenges, a group of researchers incorporated different mechanical dispersion techniques in their studies, namely ultrasonication, high shear stress, calendaring process, ball-milling, stirring, and extrusion [13,29].

### 3.1 Melt mixing

**Fig. 3:**
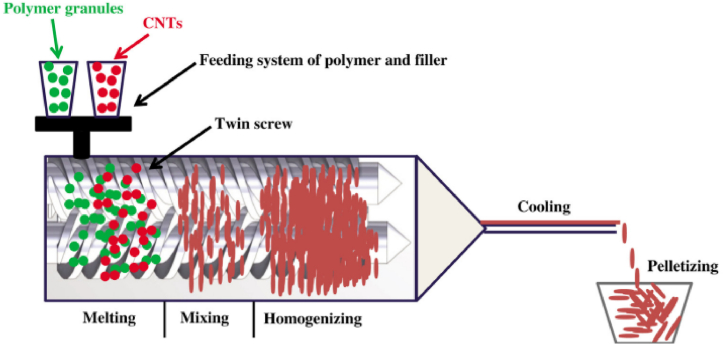
Schematic representation of a twin-screw extruder for the melt mixing of CNT/polymer composites [38].

Melt mixing is a commonly employed technique in the fabrication of CNT polymer composites. This method involves blending CNTs with the polymer matrix at an elevated temperature, typically above the melting point of the polymer. The process utilizes high shear forces generated during mixing to disperse and incorporate the CNTs uniformly within the molten polymer. Melt mixing is particularly suitable for thermoplastic polymers, where the polymer can be melted and processed multiple times without significant degradation [23]. Melt mixing helps to ensure uniform dispersion of individual CNTs throughout the polymer matrix, thereby achieving homogeneity. This uniform distribution improves the mechanical, electrical, and thermal properties of the resulting CNT polymer composite material [38]. Fig. 3 illustrates a typical twin-screw extruder used in the melt mixing process of CNT polymer composites.

In a conducted study, nanocomposites of 1 wt% CNT-PP were created through melt mixing. However, it was observed that relying solely on melt mixing did not yield a uniform dispersion of nanotubes within the polypropylene matrix [39]. In another study, a combination of compression and twin-screw extrusion was employed to produce CNT/polyethylene composites. Despite the advantages of speed and simplicity associated with melt-processing techniques, it was found that these methods were not particularly effective in breaking up the agglomeration of CNT and achieving their proper dispersion within the polyethylene matrix [40]. Limited studies focus on optimizing the melt mixing parameters (such as temperature, mixing time, shear rate) for achieving the best dispersion and properties in the resulting composites. There is a gap in the literature addressing challenges and solutions when scaling up the melt mixing process for industrial production of CNT-polymer composites.

### 3.2 Solution mixing

**Fig. 4:**
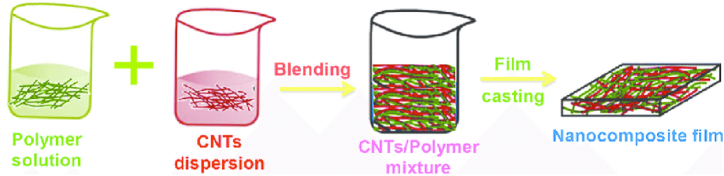
Schematic representation of solution processing method [23].

In solution mixing, CNT is dispersed in a solution that contains either the polymer precursor or a polymer compatible with the solvent. The procedure entails dissolving the polymer and subsequently dispersing CNTs within the solution as shown in Fig. 4. Once a uniform mixture is achieved, the solvent is commonly evaporated, or the polymer is precipitated, leading to the creation of a nanocomposite material [36,41,42]. Solution mixing offers advantages such as better control over the dispersion of CNT and the ability to achieve a more uniform distribution within the polymer matrix. Additionally, this method applies to a wide range of polymer types. However, challenges may arise during solvent removal, which is time-consuming compared to techniques such as melt mixing [23,41].

There is a gap regarding the environmental aspects of the solution-mixing process, including solvent choice and disposal. Limited studies focus on optimizing the solution mixing parameters such as solvent choice, concentration, and mixing time for achieving a uniform dispersion of CNTs in the polymer matrix.

### 3.3 In-situ polymerization

**Fig. 5:**
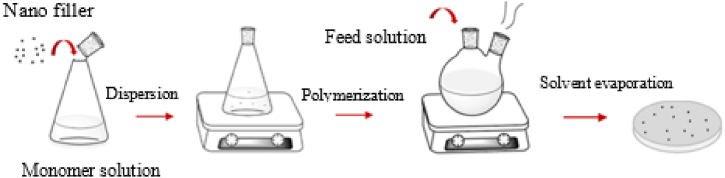
Schematic representation of in situ polymerization [42].

In-situ polymerization proves to be a highly efficient approach for enhancing the dispersion and interaction of CNTs within a polymer matrix. It serves as a practical choice for crafting composites based on polymers that are insoluble, thermally unstable, and not amenable to preparation through solution or melt processing methods [38,42]. The procedure entails dispersing CNTs into a monomer matrix, with or without the use of a solvent, followed by the execution of standard polymerization as shown in Fig. 5. This method is versatile and robust, enabling the preparation of large-area membranes featuring vertically aligned arrays of CNTs. The generated pores prevent the alignment of nanotubes from obstructing and disturbing the membranes. While this technique yields flexible, aligned CNT with a relatively high CNT density, it exhibits drawbacks such as poor CNT alignment and an increase in viscosity as the process progresses [43].

However, limited studies focus on optimizing the in-situ polymerization parameters (reaction conditions, monomer choice, initiator type) to achieve improved dispersion and enhanced properties in the resulting composites. comparative studies evaluating the pros and cons of in-situ polymerization in comparison to other methods (melt mixing, solution mixing) for CNT dispersion and composite formation is limited.

## 4 Filament extrusion of CNT polymer composites

Recent studies are focus on the manufacturing of filament from CNT polymer composites due to the enhanced physical, mechanical, electrical, and thermal properties. Filaments for AM, a widely used 3D-printing technology, could be improved by the addition of CNT to polymers. Extrusion is indeed, one of the most commonly employed techniques for manufacturing CNT polymer composite filaments, especially for 3D printing applications. Extrusion allows for the continuous production of filaments with a consistent diameter, which is crucial for the success of 3D printing processes. The most commonly used extruder machines used for making the filament are single and double screw extruder machine [18,44].

**Fig. 6:**
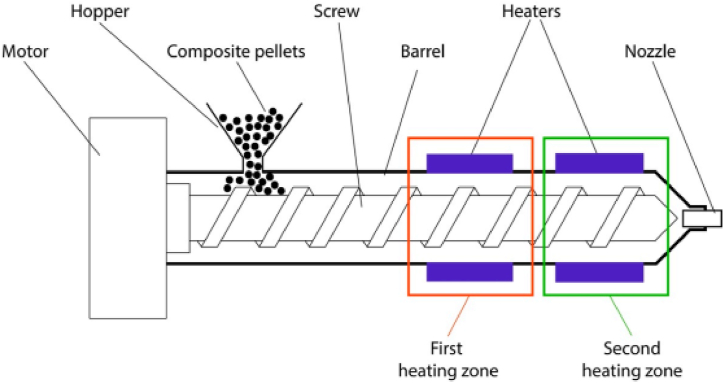
Schematic diagram of a single screw extruder for composite filaments fabrication [45].

The extrusion machine, illustrated in Fig. 6, is equipped with two independent heating zones, a rotating screw, and a die for the production of CNT polymer composite filaments used in AM through 3D printing [45]. The extrusion machine facilitates the control of the extruded filament diameter by allowing the interchange of nozzles with varying diameters. The process involves loading a mixture of polymer and CNT into the extruder, where the rotating screws catch and propel the material forward, subsequently melting it in the heated chamber. The polymer-CNT mix is then extruded through nozzles under pressure, forming semisolid filaments. These filaments are cooled by an air-fan and water system to attain the desired filament diameter. The resulting CNT-polymer composite can be employed in AM using the 3D printing technique. The exchangeable nozzles, rotating screws, and controlled heating and cooling processes are integral for ensuring the uniformity and quality of the extruded filaments for 3D printing applications [19,44].

The effectiveness of CNT dispersion through extrusion depends on several factors, including the properties of both the matrix and CNT surfaces, along with processing conditions. Screw speed and torque have a substantial impact on dispersion quality. Furthermore, the amount of time the mixture spends in the extruder is a key factor, resulting in better dispersion with prolonged residence times. The configuration of the screw is another processing parameter that influences how well CNTs are dispersed within the matrix [46]. While extrusion is commonly used to disperse CNTs in thermoplastic polymers, it has been reported that CNT dispersion in thermoset polymers, such as epoxy, can also be achieved through a method known as reactive extrusion.

Based on the literature review, investigations were conducted on ABS/CNT composite filaments with filler fractions of 0.99, 1.96, 4.76, and 9.09 wt% using a 1.7 mm nozzle diameter. The findings indicate that lower extrusion temperatures led to insufficient flow of the melted matrix material, resulting in extruder nozzle clogging and the production of heterogeneous filaments. Conversely, higher extrusion temperatures caused the material to flow too rapidly, making it challenging to achieve a consistent filament diameter [45]. In another study, the production of optimal ABS–MWCNT composite filaments for FDM through 3D printing was successfully achieved using a single screw extruder. The MWCNT contents ranged from 0.5 % to 4 % by weight. The mechanical properties of the 3D-printed samples varied, showing dependence on the MWCNT loading content in the composite filament. The study determined that the optimal MWCNT fraction for the fused deposition modeling (FDM) process was 2.0 wt%, resulting in samples with favorable mechanical and thermal properties suitable for practical applications [47].

In summary, the optimization of the extrusion process has proven instrumental in achieving improved filament quality. Rigorous testing and characterization are necessary to confirm the mechanical, thermal, and electrical properties of the CNT-polymer composite filament to get a quality 3D print. Furthermore, careful consideration should be given to the compatibility of the composite with the specific 3D printing technology employed. This comprehensive approach ensures the production of high-quality and application-specific CNT-polymer composites.

## 5 Additive manufacturing of CNT polymer composite filament

Currently, additive manufacturing (AM) is extensively utilized for its precision in creating intricate models. However, the structural anisotropy resulting from 3D printing approaches may impose limitations on robust applications. One potential solution to address the inferior properties of materials produced through 3D printing compared to conventionally manufactured counterparts involves incorporating nanoparticles, such as CNTs, which exhibit remarkable mechanical, electrical, and thermal properties [19].

Until now, numerous conductive nanoparticles have been employed in 3D printing. Nevertheless, limited research has concentrated on creating nanocomposite filament feedstock specifically designed for FDM using 3D printing. Enhancements to filaments utilized in FDM, a widely adopted 3D-printing technology, can be achieved through the incorporation of nanofillers. Notably, achieving uniform dispersion of CNT in a polymer matrix opens avenues for producing 3D-printed components applicable across diverse sectors, including aerospace, automotive, electronic sensors, circuits, and micro batteries [44,48].

**Fig. 7:**
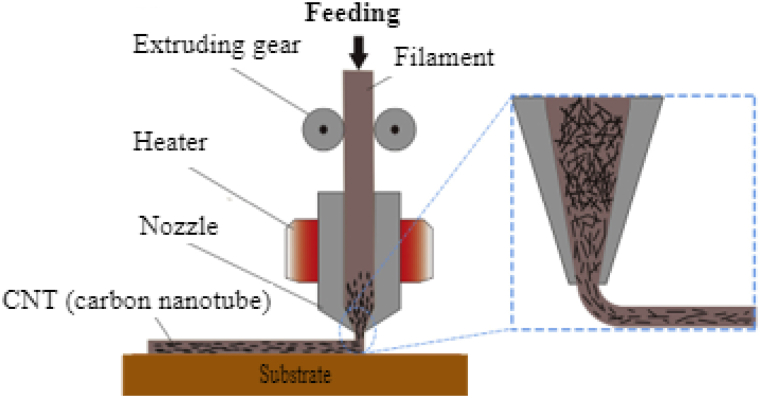
Schematic diagram of 3D printed CNTs polymer composite materials [49].

The filaments undergo melting into a semi-liquid state at the nozzle and are extruded layer by layer onto the build platform. These layers fuse and subsequently solidify into the final parts, as illustrated in Fig. 7. The quality of the printed components can be managed by adjusting various printing parameters, including layer thickness, printing orientation, raster width, raster angle, and air gap. In contrast to conventional production methods, the Fused Deposition Modeling (FDM) method facilitates the creation of intricate geometries with high precision, at a reasonable cost, ensuring maximum material efficiency, design flexibility, and opportunities for personalized customization. AM leads to a reduction in material waste by around 90 %, as opposed to subtractive manufacturing methods [49,50]. Limited studies focus on optimizing the AM parameters specifically for CNT-polymer composite filaments such as printing speed, layer thickness, and nozzle temperature.

## 6 Micromechanical modeling of CNT polymer composite

Micromechanical modeling is a crucial tool for understanding and predicting the behavior of CNT polymer composites. By developing microscopic virtual representations of these materials, researchers can examine the interactions between the polymer matrix, CNTs, and their interface under various circumstances. Recent studies have made significant advancements in this field, utilizing different approaches to estimate the behavior of these materials under different loading conditions [[51], [52], [53], [54]].

The effective elastic characteristics of CNT polymer composites can be predicted using a multiscale modeling framework that integrates micromechanical analysis and molecular dynamics simulations. The model accounts for the influence of CNT waviness and the quality of the CNT-matrix interface on the overall properties of the composite [51]. Another study employed a temperature-dependent percolation micromechanical model that integrates the effects of temperature on the percolation threshold and the electrical conductivity of CNT-polymer composites. This approach enables the prediction of electrical conductivity and piezoresistive sensitivity by considering the distribution and connectivity of CNTs within the polymer matrix as a function of temperature [52]. The creep performance of CNT polymer composites was examined in a different study using a multi-stage hierarchical micromechanical model. The model predicts the impact of CNT agglomeration on the mechanical properties of the composite materials and focuses on the viscoelastic behavior of the interphase area surrounding the CNTs. Overall, the results indicate that it is critical to consider the interaction, CNT dispersion, and agglomeration effects for improved predictions of creep performance in CNT polymer nanocomposites [53]. On the other hand, the thermal conductivity of CNT shape memory polymer was predicted using a unit cell micromechanical model. This model helps to analyze the thermal properties within the composite material by considering the content, arrangement, and interaction of the CNTs within the polymer matrix [54]. Moreover, the researchers forecast the electrical conductivity of CNT polymer composites in the presence of an external magnetic field using a model based on percolation theory. This model predicts the percolation threshold, as well as the effects of the magnetic field on the charge alignment and mobility of CNT [55].

These studies demonstrate the value of micromechanical modeling for understanding and predicting the behavior of carbon CNT polymer composites. These models considered the effects of multiple parameters, including CNT dispersion, aspect ratio, and interfacial properties, and provided valuable insights into the design and optimization of these advanced materials.

## 7 Properties of CNT polymer composites

The properties of CNT-polymer composites vary based on factors such as the polymer type, CNT weight fraction, processing conditions, and other parameters. These composites display a spectrum of distinctive and desirable characteristics, including a lightweight nature, improved mechanical strength, and electrical and thermal conductivity, among others [8,9,13,23,[56], [57], [58], [59]]. Understanding and optimizing these properties enable the design and fabrication of advanced materials with a wide range of applications across industries such as aerospace, automotive, electronics, biomedical, materials science, and other applications.

### 7.1 Microstructure of CNT polymer composites

The microstructure of CNT polymer composites determines their performance of the composite. For instance, the mechanical properties are greatly influenced by CNT dispersion, interfacial bonding, and crystallization behavior; perfect control results in increased strength, stiffness, and toughness [60]. For electrical conductivity, careful control of CNT dispersion, aspect ratio, percolation threshold, and interfacial interactions is essential to form efficient conductive networks [61]. Similarly, good thermal performance relies on proper CNT dispersion, aspect ratio, interfacial bonding, and percolation threshold to create efficient heat transfer pathways [62].

**Fig. 8:**
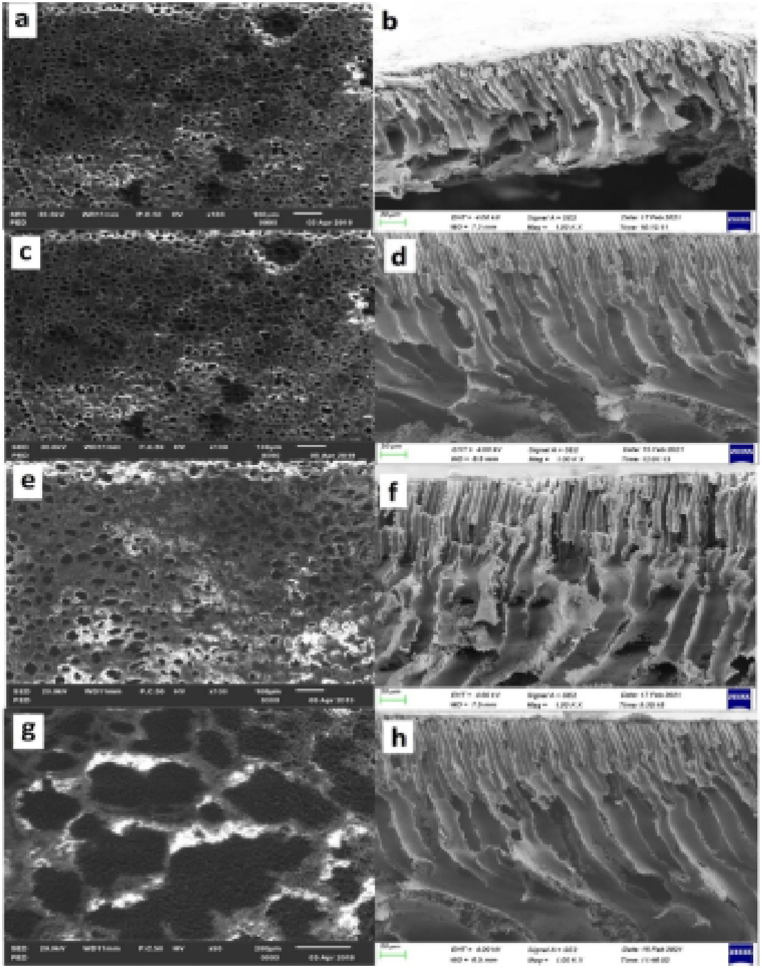
SEM images of PES/CNT membranes with varying wt.% CNT (a) surface at 2.5 wt% CNT (b) cross-section at 2.5 wt% (c) surface at 5 wt% (d) cross-section at 5 wt% (e) surface at 7.5 wt% (f) cross section at 7.5 wt % (g) surface SEM at 10 wt% h) cross-section at 10 wt% [63].

Fig. 8 illustrates the surface and cross-sectional morphology of the PES membrane with varying loadings of CNTs. Here, Fig. 8(a) and (b) depict the surface and cross-sectional morphology of the PES/2.5 wt% CNT, while Fig. 8(c) and (d) show these characteristics for the PES/5 wt% CNT. Fig. 8(e) and (f) illustrate the surface and cross-sectional views of the PES/7.5 wt% CNT, and Fig. 8(g) and (h) present the same for the PES/10 wt% CNT. Adding CNTs creates larger and more dispersed pores in the membrane compared to pure PES membranes. This effect is most pronounced with a 5–7.5 wt% CNT loading. At the highest loading (10 wt%), the pores become smaller, this is likely due to CNT clumping (agglomeration) which hinders pore formation. Lower CNT loadings create a less viscous solution, allowing for faster solvent exchange and larger pore formation. Higher loadings increase solution viscosity and CNT agglomeration, hindering solvent exchange and leading to smaller pores [63].

### 7.2 Physical properties of CNT polymer composites

Carbon nanotube-polymer composites offer a range of enhanced physical properties due to the unique characteristics of both CNTs and polymers. Density stands out as a crucial parameter in evaluating the physical properties of these composites. Due to the inherently lightweight nature of polymers, the incorporation of CNTs results in lightweight CNT-polymer composites [[64], [[65], [66]].

**Fig. 9:**
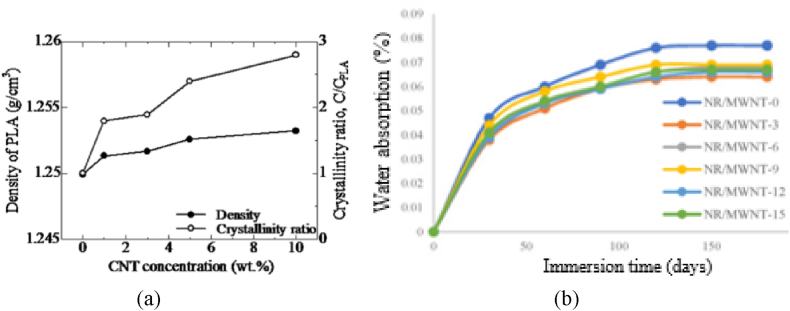
Physical properties (a) density and crystallinity ratio of CNT/PLA composite [67] and (b) Water absorption of MWCNT/NR [68].

The researcher examined ABS filaments containing varying amounts of CNTs, ranging from 1 % to 8 % by weight. The density of MWCNT/ABS filament exhibits nearly linear growth with an increasing fraction of MWCNT, reaching 1.081 $g/{\text{cm}}^{3}$ at 8 wt% MWCNT [64]. An additional investigation utilizing CNT-filled ABS discovered that raising the CNT content from 5 % to 10 % resulted in a nearly linear rise in the material's density. The final density was influenced by how the composite material was manufactured. For composites created by compression molding, adding 10 % MWCNTs increased density to 1.083 $g/{\text{cm}}^{3}$, while 10 % of SWCNTs raised it to 1.086 $g/{\text{cm}}^{3}$. When the composite was made filament extrusion, 10 % MWCNTs resulted in a density of 1.094 $g/{\text{cm}}^{3}$, and 10 % SWCNTs gave a density of 1.076 $g/{\text{cm}}^{3}$ [65]. In another research, the density of CNT epoxy was measured for MWCNT ranging from 1 % to 20 % of the total volume. As expected, adding more MWCNT caused the material to become denser. When CNT content increased from 0 % to 20 % the density decreased from 1.40 $g/{\text{cm}}^{3}$ to 1.32 $g/{\text{cm}}^{3}$, which is an increase of roughly 6 % [66]. In another study, the density and crystallinity of the PLA were greatly increased with the increase in the CNT concentration [67] as shown in Fig. 9 (a). The environmental stability of the MWCNT/NR composite was checked through a water absorption test as illustrated in Fig. 9 (b), and water absorption stabilized after an initial 30-day diffusion period [68].

### 7.3 Mechanical properties of CNT polymer composites

**Fig. 10:**
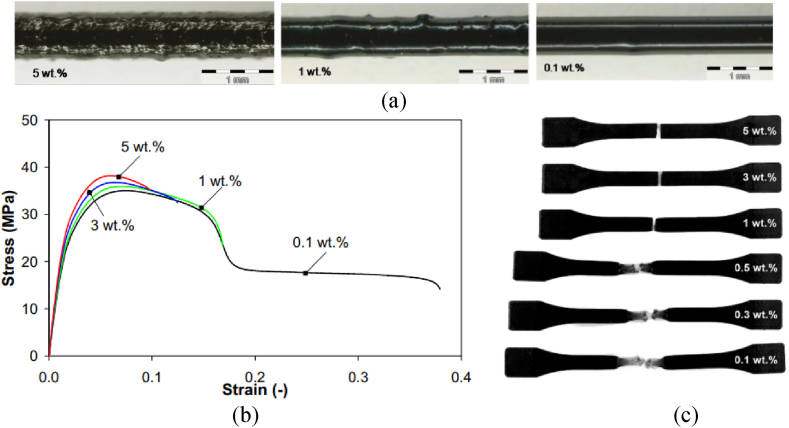
(a) Extrudate filaments, (b) stress-strain (c) injection-molded samples after testing for the PP/MWCNT at different MWCNT wt.% [72].

Numerous recent experimental studies, as indicated in Table 2, have demonstrated that the incorporation of a small quantity of CNT into polymers can lead to notable enhancements in the mechanical properties of CNT polymer composites. The addition of CNT to polymers holds the promise of delivering substantial improvements in terms of strength, stiffness, and toughness for these composite materials [45,47,57,[69], [70], [71]]. Fig. 9 illustrates the mechanical properties of MWCNT/PP composites and their corresponding test samples [72]. The amount of CNTs used determines how the CNTs are arranged within the material, as Fig. 10 (a) illustrates. As the content of MWCNTs increases, the material's ability to withstand stress and young modulus increases while its strain decreases, as shown in Fig. 10 (b). Additionally, the fracture behavior of the sample is significantly influenced by the content of MWCNTs, as shown in Fig. 10 (c).

**Table 2:** Mechanical properties of CNT-polymer composites reported by various researchers.

Materials	Mechanical properties	Ref.
MWCNT/ABS	Filaments containing 0.99, 1.96, 4.76, and 9.09 wt% MWCNTs were produced using a 1.7 mm nozzle. While small amounts of CNTs weakened the printed parts, a 9.09 % CNT concentration significantly increased tensile strength by 12.6 %.	[45]
MWCNT/ABS	Tensile tests were conducted with 0.5, 1, 1.5, 2, 3, and 4 wt% MWCNT. Adding MWCNTs improved the tensile strength of ABS samples, increasing from 31.43 MPa (pure ABS) to 44.57 MPa at 2.0 wt% of MWCNTs—a 41.81 % increase. However, no further improvement was observed above 3.0 wt %. The material is brittle at low MWCN and its elongation increased at higher concentrations of MWCNT (1.5 wt% and above).	[47]
MWCNT/PP MWCNT/HDPE	The experiment was made at 0 (neat polymer), 24, 39, and 47 vol% of MWCNT. Increasing MWCNT content from 0 % to 47 % in HDPE improved material strength, showing greater mechanical reinforcement in the HDPE composites as the CNT concentration rose.	[57]
MWCNT/Epoxy	Incorporating 1 and 4 wt% MWCNT into the epoxy significantly boosts mechanical properties. The Young's modulus and yield strength of the composite double with 1 wt% MWCNT and quadrable with 4 wt% MWCNT.	[69]
MWCNT/PLA	Adding 5 % MWCNT to 3D-printed PLA scaffolds and compressing it created a strong, interconnected structure. This improved Young's modulus and bending strength by 101 % and 43 %, respectively, compared to PLA with randomly distributed CNTs.	[70]
MWCNT/Epoxy	CNT/Epoxy composite was made at 0, 0.5, 1, 2, and 4 wt% MWCNT. Increasing MWCNT content from 0 % to 2 % improved the composite's performance in tension, shear, and flatwise tests.	[71]

Though the mechanical properties of CNT polymer composite depend on the CNT content, polymer type, and manufacturing method, limited research done on optimizing the even distribution of CNT within polymer materials, this is crucial for improving the mechanical properties of the composite. New or improved methods are needed to achieve a more uniform dispersion of CNTs and unlock the full potential of these materials. Unlocking the full potential of these materials requires new or improved methods to create a more uniform dispersion of CNTs.

### 7.4 Electrical properties of CNT polymer composite

**Fig. 11:**
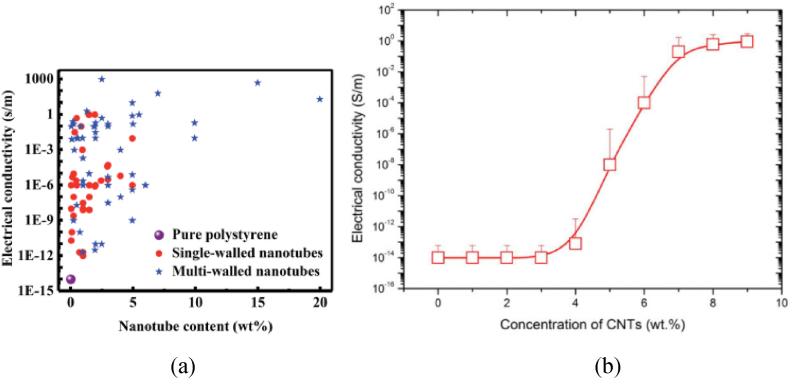
Electrical conductivity (a) pure polystyrene, SWCNT–polystyrene, and MWCNT– polystyrene composites [74], (b) CNT/PDMS composite films with varied CNT concentration [75].

Carbon nanotube polymer composites exhibit exceptional electrical properties due to the unique characteristics of CNTs. The high aspect ratio of CNTs allows them to form a conductive network within a polymer matrix, even though the matrix itself is typically an insulator. This conductive network enhances the electrical conductivity of the composite. CNTs are useful additions in polymer composites for applications needing better electrical capabilities because of their special structure and superior electrical conductivity. CNTs, with their distinctive structure, exhibit the capability to carry significantly higher currents compared to other materials [45,49,64,65,69,73]. As indicated in Fig. 10, the electrical conductivity of the CNT polymer composite increases with an increase in CNT loading. The electrical properties of CNT polystyrene composite change as types of CNT, CNT loading, and manufacturing methods change [74] as shown in Fig. 11 (a). The electrical conductivity of the CNT/PDMS composite increases with an increase in CNT concentrations [75] as indicated in Fig. 11 (b). As Table 3 shows, efforts are being made to customize these qualities to the precise requirements of certain applications, to improve the electrical conductivity of CNT polymer composite.

**Table 3:** Electrical properties of CNT-polymer composites reported by various researchers.

Materials	Electrical properties	Ref.
MWCNT/ABS	MWCNT/ABS filament was made at CNT 0.99, 1.96, 4.76, and 9.09 wt% MWCNT using a 1.7 mm nozzle. Filament made with 4.76 and 9.09 wt% MWCNT exhibited average resistivities of 2.5 Ω. m and 0.15 Ω. m, respectively, under 12 V. As the voltage increased from 2 to 18 V, the resistance decreased by 83 % for the 4.76 wt% MWCNT composite and 88 % for the 9.09 wt% MWCNT composite.	[45]
MWCNT/TPI	CNT/TPI was produced 1, 3, 5, 7, and 9 wt% MWCNT. The resistivity of both filaments and 3D-printed specimens gradually decreased with the increase in the content of CNTs in the TPI resin matrix.	[49]
MWCNT/ABS	MWCNT/ABS filament was made with 1, 2, 4, 6, and 8 wt % MWCNT. Pure ABS is an insulator (10^15^Ω. cm resistivity) and adding up to 2 wt% CNT remains insulating. The electrical resistivity decreases to 11 Ω. cm, 4.1 Ω. cm, and 1.8 Ω. cm for 4, 6, and 8 wt%, respectively.	[64]
CNT/ABS	Both SWCNT and MWCNT significantly improved conductivity in ABS when increased to 5–10 %. Resistivity dropped to 0.19 Ω cm for SWCNT and 0.65 Ω cm for MWCNT at 10 % concentration.	[65]
MWCNT/Epoxy	0.5 wt% MWCNT to epoxy remains nonconductor. However, increasing MWCNT to 1 and 4 wt% made the material conductive with the values of $10^{-3}\;\text{and}\;6\times 10^{-2}\;S/\text{cm}$, respectively. The critical point for conductivity is between 0.5 % and 1 % CNT.	[69]
MWCNT/ABS	MWCNT/ABS composites with 1, 3, 5, 7, and 10 wt% MWCNTs were prepared. While pure ABS is an insulator (with $10^{-12}$ S/cm conductivity), adding 10 wt% CNT increases its conductivity to $10^{-5}$ S/cm.	[73]

The primary gaps in the literature are in the areas of advanced characterization, long-term durability, understanding the influences of conductivity, investigating new CNT kinds, and getting better electrical properties of CNT polymer composites with uneven CNT distribution. The electrical properties of CNT polymer composites can be enhanced through optimized CNT-polymer composite fabrication, leading to applications in energy, sensing, electronics, and specialized materials.

### 7.5 Thermal property of CNT polymer composite

The enhanced thermal conductivity of CNTs makes them attractive options for improving the thermal properties of polymer composites. The thermal conductivity, thermal stability, heat dissipation, and thermal expansion of composites can all be markedly improved when CNTs are added to the polymer matrix. The unique one-dimensional structure of CNTs allows for efficient heat transfer along their length. The thermal performance of CNT polymer composites makes them suitable for various applications, including thermal interface materials, heat sinks, and components in electronic devices where effective heat management is essential.

**Fig. 12:**
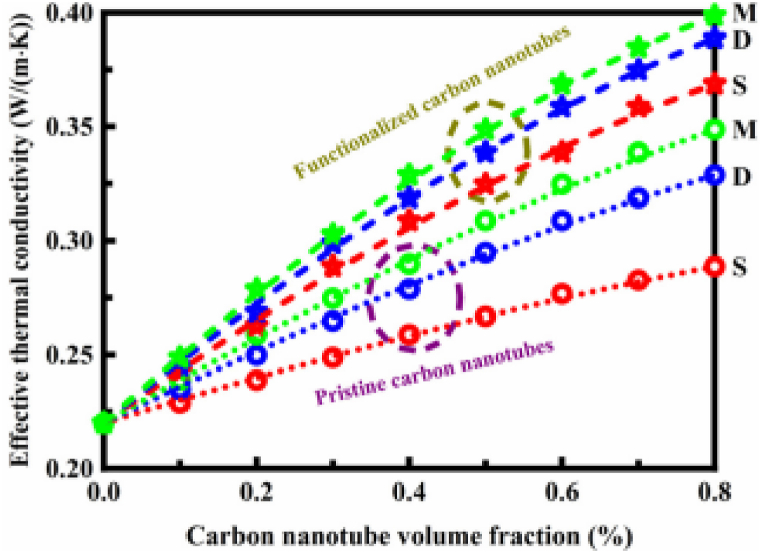
Effective thermal conductivity of the CNT polymer materials as a function of CNT concentration. S: single-walled CNT, D: double-walled CNT, and M: multi-walled CNT [76].

The thermal conductivity of a polymer can be markedly enhanced by adding small amounts of CNT as shown in Fig. 12. Adding 0.8 % of hydroxyl-functionalized MWCNT, in particular, increased heat conductivity by more than 80 % [76].

Researchers are continually exploring ways to further enhance and tailor the thermal properties of these composites for specific industrial applications. Table 4 presents an overview of the thermal properties of CNT polymer composites, compiled from a review of various research studies.

**Table 4:** Thermal properties of CNT-polymer composites reported by various researchers.

Materials	Thermal properties	Ref.
MWCNT/PP MWCNT/HDPE	The composite was made at 0, 24, 39, and 47 vol% MWCNT. Increasing MWCNT content in both PP and HDPE matrices significantly improved thermal conductivity. At the highest MWCNT loading, thermal conductivity increased by 150.1 % (k = 0.564 W/m K) for PP and 79 % (k = 0.859 W/m K) for HDPE compared to the neat polymers.	[57]
MWCNT/ABS	Filaments were produced at 1, 2, 4, 6, and 8 wt% MWCNT. The composite with 8 wt% MWCNT showed the greatest reduction in the coefficient of thermal expansion, decreasing from $79.6\times 10^{-6}\;K^{-1}$ for ABS to $52.3\times 10^{-6}\;K^{-1}$ at room temperature.	[64]
SWNT/PMMA	Incorporating SWNT into a PMMA matrix leads to an enhancement in the thermal conductivity of composite films. Specifically, at a 7 % SWNT loading, the thermal conductivity of the composites increases by 55 %.	[77]
SWCNT/PVDF	The experiment was conducted at 0, 5, 10, 19, 29, 39, and 49 vol% SWCNT. Thermal conductivity increased from 0.233 to 0.537 W/m K, with a 130 % rise at 49 vol% SWCNT, but remained below the 1 W/m K threshold for effective heat sinks. Conductivity also rose with temperature (25–150 °C). The composite's melting temperature (174 ± 8 °C) and enthalpy of fusion were unaffected by SWCNT content.	[78]

## 8 Application areas of CNT polymer composite

The unique structural, electrical, and mechanical properties of CNTs, made CNT polymer composites a promising candidate for a broad advantage in the applications of sensors, electronic devices, energy storage, biomedical, and many more. The potential and current applications of CNT polymer composites include electronics, automobiles, textiles, aerospace, sports equipment, sensors, energy storage, and others due to their high durability, high strength, lightweight, design and process flexibility [8,13,59].

### 8.1 Structural materials

In the context of structural materials, CNT polymer composites are utilized to enhance the mechanical and structural properties of various materials. The incorporation of CNTs into polymers can result in composites with improved strength, stiffness, and other desirable characteristics. The application areas within the realm of structural materials are aerospace components, automotive parts, construction materials, sports equipment, marine applications, military and defense, infrastructure repair and reinforcement, advanced structural materials, civil engineering applications, etc. [10,13,22]. In addition, the remarkable property-to-weight ratio of CNT polymer composites makes them ideal candidates for structural applications. To use successfully CNT polymer composites in engineering applications, it is significant to predict the structural responses of this new class of nanocomposites under different loading conditions [8]. Nowadays, fiber-reinforced composites such as glass, carbon, and hybrid fabrics are commonly used in structural applications. However, CNT polymer composites exhibit superior strength compared to these materials. Studies indicate that CNTs significantly boost tensile performance, with CNT composites demonstrating a 69 % increase in tensile strength over carbon fiber composites, achieving a heightened strength-to-weight ratio and increased stiffness-to-weight ratio [79]. The unique combination of strength, flexibility, and lightweight properties of CNT polymer composites makes them promising materials for advancing structural applications across different industries. Ongoing research continues to explore and optimize these materials for specific structural requirements.

### 8.2 Aerospace

CNT polymer composites can be employed in the aerospace industry to manufacture lightweight and high-strength components for aircraft and spacecraft. The enhanced mechanical properties can contribute to fuel efficiency and overall performance. Application areas of CNT polymer composites in the aerospace sector aircraft components, spacecraft and satellites, rocket components, unmanned aerial vehicles, aircraft interiors, antenna structures, thermal protection systems, airframe components, aerospace coatings, and aerospace research and development [10,16,22]. The use of CNT polymer composites in aerospace applications is a dynamic field, and ongoing research aims to further optimize these materials for specific aerospace requirements, ensuring safety, reliability, and performance. CNTs hold promising prospects in aerospace applications, such as reducing vehicle weight, enhancing self-healing properties, advancing energy production, improving controls with superior tolerance capabilities, and upgrading thermal protection [11,13]. In aerospace applications, CNT, carbon fiber, and glass fiber polymer composites are utilized. Adding CNT to carbon fiber polymer led to enhancements in tensile strength by 27.5 %, 53.25 %, and 40 %, respectively [80]. Airframes made with CNT polymer composites instead of aluminum can reduce structural mass by an average of 14.05 %, which will extend flight range by 13.2 % and reduce fuel consumption by 9.8 %. Aluminum, due to its lower electron density and the production of secondary particles, exhibits reduced attenuation properties. Meanwhile, CNT provides excellent resistance to air oxidation compared to aluminum [81].

**Fig. 13:**
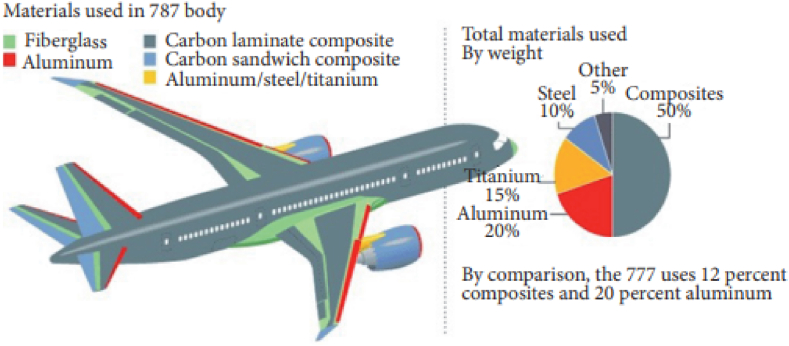
The overall distribution of composite materials used in Boeing 787 aircraft [83].

The Boeing 787 was the pioneering commercial aircraft to incorporate a main structure with 50 % carbon-reinforced composite material. The details are highlighted in Fig. 13, illustrating the material distribution as 50 % carbon composite, 20 % aluminum, 15 % titanium, 10 % steel, and 5 % other materials. This integration led to a significant 20 % reduction in weight due to the adoption of composite material. In comparison, the Boeing 777's structure is primarily composed of 50 % aluminum, with a mere 12 % attributed to composite material [82]. Substituting the existing 50 % composite materials in the Boeing 787 with CNT polymer composites offers significant advantages over carbon fiber-reinforced composites in terms of mechanical strength, weight efficiency, electrical and thermal conductivity, and compatibility with advanced manufacturing techniques. These benefits position CNT polymer composites as a transformative solution for aerospace applications, potentially leading to enhanced performance and efficiency in aircraft design and operation.

### 8.3 Automotive

**Fig. 14:**
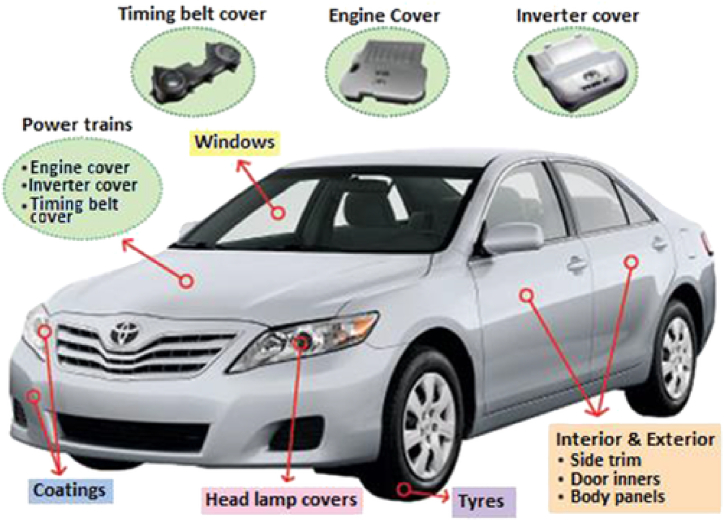
The use of CNT/polymer in various parts of the car [84].

The application of CNT/polymer composite materials in the automotive industry is an area of active research and development. These composites leverage the unique properties of CNTs, such as high strength, lightweight, and electrical conductivity, when combined with polymers. In the automotive industry, these composites can be employed to reduce the weight of vehicles, improving fuel efficiency and overall performance. The potential applications of CNT polymer composites in automotive, as indicated in Fig. 14, for structural components for body panels and chassis components, interior components for seats and interiors, bumpers and fenders due to its impact resistance, tire reinforcement, and anti-corrosion coatings for the exterior surfaces of vehicles [84,85]. CNT polymer composite has the potential used in engines, powertrain systems, braking systems, emission systems, catalyst converters, body and frame components, paints and coatings, lubrication, tires, as well as electrical and electronic equipment [84].

Applications of CNTs/polymer composites in the automotive sector encompass technological progress in existing systems, notably in the realms of body components, electrical systems, and engine parts. Incorporating CNTs, combined with fiberglass reinforcement in epoxy composites, has the potential to elevate strength and impact energy by 60 % and 30 %, respectively. As a result, this enhancement could play a pivotal role in diminishing fuel consumption and greenhouse gas emissions by 16 % and 26 %, respectively [22]. CNT fillers prove to be efficient at lower concentrations (0.2 wt%) in polymeric composites as they can markedly improve dimensional and thermal stability while also decreasing overall weight. Reducing vehicle weight by 25 % could lead to a significant decline in crude oil barrel consumption, potentially saving up to 250 million barrels annually [13].

### 8.4 Construction

Incorporation of CNT polymer enhances the structural integrity and durability of the materials, making them suitable for use in bridges, buildings, and other infrastructure [86,87]. CNT polymer composites have shown potential applications in the construction industry, offering a range of desirable properties that can enhance the performance of various building materials. It can enhance the mechanical strength and durability of construction materials like concrete, making them suitable for infrastructure applications [23,88]. The use of CNTs in construction enhanced flexural and tensile strengths by up to 50 % and compressive strength by up to 30 %. The optimal CNT content to achieve these improvements is up to 0.1 % by weight of cement [89]. CNT polymer composites are incorporated into various civil engineering applications, such as the construction of bridges, tunnels, and other infrastructure, to enhance structural performance and longevity. The CNT-reinforced double beam system has large application value in tracks of railway, vibration absorbers, and cranes [58].

### 8.5 Electronics

**Fig. 15:**
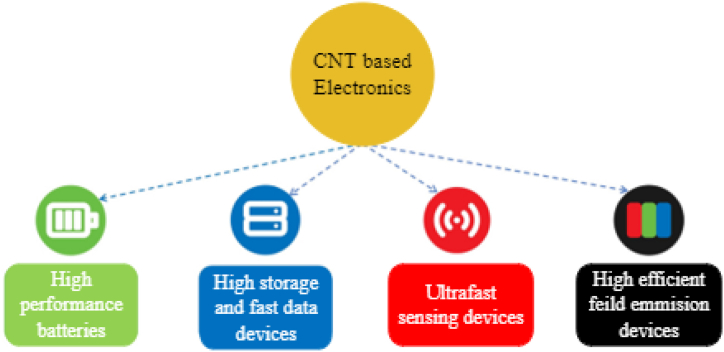
Advantages of CNT-based electronics [90].

Currently, there is a desire for superior materials to substitute existing ones, aiming for product streamlining, high-performance devices with reduced weight, and a reduction in electronic waste. This objective can be realized through the incorporation of materials based on CNTs [90], as indicated in Fig. 15. CNT polymer composites are extensively utilized in electronic applications due to their unique electrical, thermal, and mechanical properties. The use of CNT polymer composites leads to lightweight structural materials with functionalities that can be utilized in broad applications such as electronic components, micro-batteries, circuits, and electromagnetic shielding. The electronic applications of CNT polymer composites include flexible electronics, conductive polymers, sensors and detectors, field effect transistors, electromagnetic interference shielding, energy storage devices, printed electronics, thermal management, transparent conductive films, and printed circuit boards [8,10,22,23,91,92]. Aluminum and copper are commonly used materials in electronic applications. CNT guarantees an electrical conductivity of 100 MS/m, greater than aluminum and copper, which are 35 and 59.6 MS/m, respectively. Steel's electrical conductivity is 4 MS/m, nearly 69 % of copper wire. Incorporating CNT into the CU and Al results in improved conductivity [93].

### 8.6 Energy storage

Today the inclusion of CNT into polymer matrix is receiving a lot of interest for energy-storage systems. These nanotubes exhibit noteworthy characteristics such as substantial surface areas, high aspect ratios, excellent conductivity, electrochemical stability, and low mass densities, making them particularly well-suited for applications in energy storage [91,94]. Some notable applications in the field of energy storage are in supercapacitors, batteries, fuel cells, energy harvesting devices, lithium-ion batteries, hybrid energy storage systems, flexible energy storage, electrochemical capacitors, and thermal energy storage [12,56]. The application of CNT polymer composites in energy storage reflects their potential to address the growing need for advanced materials that can enhance the performance, efficiency, and environmental sustainability of energy storage devices. CNTs are making strides in the field of renewable energy, with companies working on the development of CNT antennas designed to capture as much as 90 % of solar energy. This is achieved by utilizing smaller sizes that align with the wavelength of sunlight [94]. CNTs are promising for supercapacitors due to their excellent electrical properties and structure. Comparisons were made on CNTs/graphene, CNTs/metal, and CNTs/polymer electrodes, and the results show that CNT/metal supercapacitors have a higher energy density (about 80 Wh/kg), while CNT/graphene supercapacitors excel in power density (9 kW/kg) [95]. Compared to glass fiber (GF) epoxy composite in thermal storage applications, the thermal conductivity of MWCNT-GF epoxy composite increased by more than 40 % [96]. In the other investigation CNT epoxy composites exhibit about 67 % higher thermal conductivity compared to GF- epoxy composites [97].

### 8.7 Biomedical

**Fig. 16:**
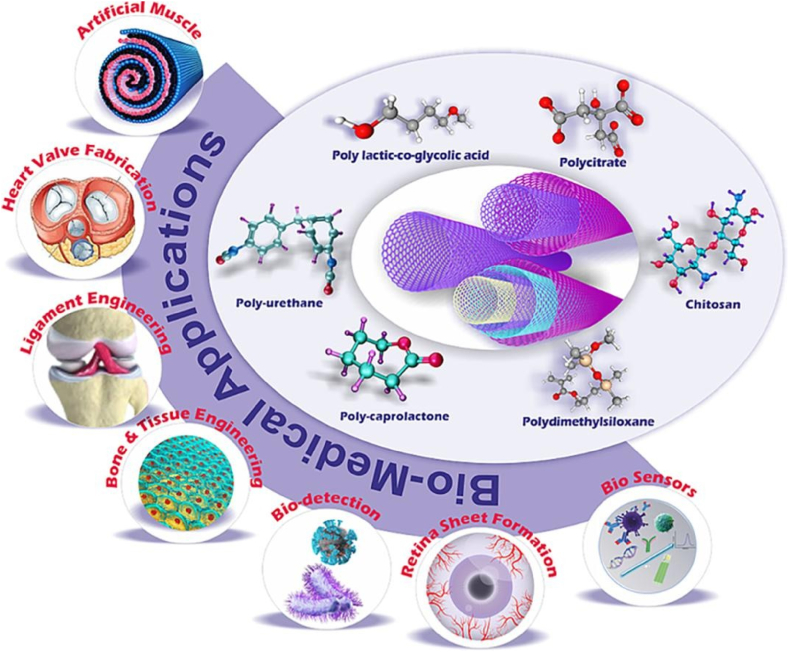
CNT polymer composite for biomedical application [98].

Nowadays, CNT polymer composites show a promising potential for biomedical applications. The unique properties of CNTs, such as their high aspect ratio, excellent mechanical strength, and electrical conductivity, make them attractive for various biomedical purposes and may become useful scaffold materials for tissue engineering. Utilizing CNTs to reinforce scaffolds has been proposed as an effective strategy for the development of engineering materials aimed at tissue regeneration [7,68,98]. Fig. 16 illustrates the potential use of CNT polymer composites in biomedical applications. Compared to polyurethane, a material frequently used for prosthetic feet, CNT natural rubber composites containing 1 wt% CNTs demonstrated a 25 % higher tensile strength. From the findings, adding 1 wt% CNTs enhanced the dimensional stability and heat resistance by 20 % and 15 %, respectively, compared to natural rubber [68].

### 8.8 Other applications

**Fig. 17:**
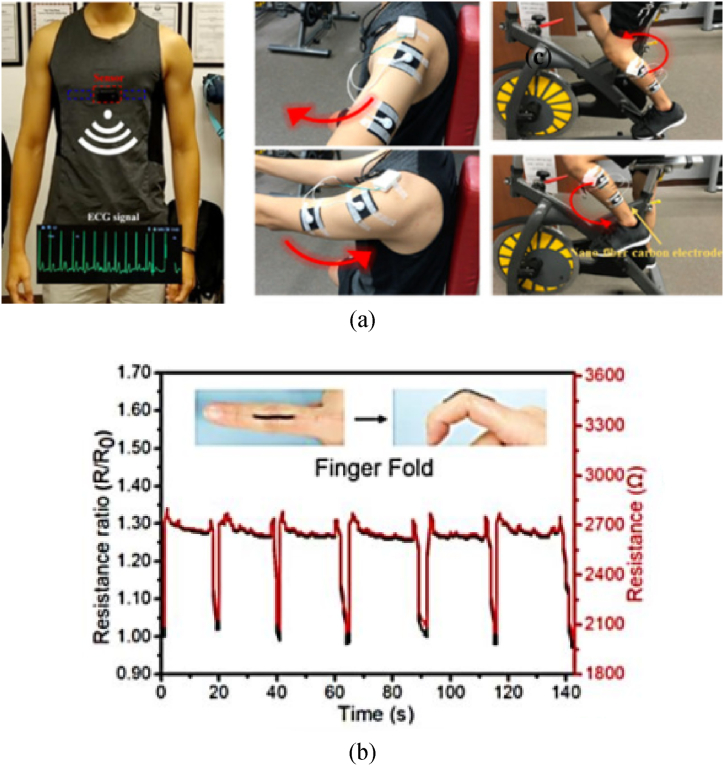
(a) The application of the nanofiber-carbon electrode in measuring the ECG and EMG signals [99], (b) applications of polyurethane/CNT helical yarn as a strain sensor for monitoring resistance changes during finger folding [100].

The unique set of properties possessed by CNTs has ushered in a new era of improved multifunctional materials. Currently, CNT polymer composites find their most widespread applications in sensors, actuators, supercapacitors, transistors, and thermoelectric devices [37]. This CNT polymer composite is also used in sporting goods, wind turbine blades, environmental remediation, catalysts, anticorrosion applications, etc. [23,91]. CNT polymers have excellent selectivity, sensitivity, and responsiveness, making them excellent sensor materials. They are very useful for detecting a wide range of analytes due to their distinct electron and charge transport characteristics. CNTs are used in gas sensors to identify gases by adsorption, connecting, and bonding. These materials are found in sensors that track biophysical signals, such as the electromyogram (EMG) and electrocardiogram (ECG), as Fig. 17 (a) illustrates. Studies reveal that using CNT electrodes enhances skin contact and leads to higher signal quality over an extended period of use. CNTs have fine stability and sensitivity for target analytes in multifaceted sensing fields including strain sensing. As shown in Fig. 17 (b), CNTs increase the stability and sensitivity of strain sensors, which makes them very useful for this application. Additionally, because of their enormous surface area, conductivity, and electrocatalytic activity, CNT-polymer composites exhibit exceptional bio-sensitivity and selectivity for the detection of biological and chemical compounds [99,100].

Extensive research has been done on the use of CNT in strain sensors. Flexible, high-performance strain sensors were created by layering CNTs on polyurethane sponges. These sensors accurately detect stretching and compression, and the best performance is achieved by combining CNTs and graphene. They excel in sensitivity, responsiveness, and durability, enabling applications in monitoring human movement and weight distribution [101]. A novel kind of extremely sensitive strain sensor was made using 3D printing a mixture of CNTs and plastic. The resulting sensor was robust to frequent use and capable of measuring significant amounts of stretching and shrinking with accuracy. The sensor was suitable for monitoring human activity and may find application in wearable technology and sophisticated robots due to its ability to detect even minute motions and vibrations [102]. Plastic, CNTs, and graphene oxide were combined to create a strain sensor. The developed sensor could be used to track human movement since it measures a broad range of stretching correctly [103]. A blend of CNTs and plastic has been applied to a flexible material to produce a highly elastic and sensitive strain sensor. The stretching and shrinking can be consistently measured by this sensor, and it can function even after being stretched and released repeatedly. Wearable technology and sophisticated robotics can benefit from the sensor's ability to detect minute motions and its insensitivity to light [104]. A mixture of CNTs and graphene flakes was 3D printed onto a plastic substance to produce a sensor. This sensor can identify pressure and stretching forces as well as the pressure's direction. It was discovered that 75 % CNT and 25 % graphene flakes were the optimum material combination, producing a sensor with excellent sensitivity, accuracy, and durability. According to this study, utilizing both of these materials enhances the sensor's performance over using only one [105]. CNT and plastic were combined to create the sensors by 3D printing. The sensors could measure pressure precisely and were made to withstand severe stretching without breaking. The sensitivity of the sensor was influenced by the amount of CNT; higher CNT produced better results. These sensors help track human activity because they can accurately detect different kinds of pressure and movement [106].

The efficiency of solar cells is increased these days by the usage of CNT polymer materials. Since CNTs improve conductivity and charge transport channels inside the polymer matrix, CNT-based composites can increase the power conversion efficiency of organic solar cells (OSCs). Introducing CNTs into OSCs increased efficiency by up to 14 % [107]. Moreover, CNT polymer composites are used in microbial applications due to their enhanced electrical conductivity, high surface area, mechanical strength, chemical stability, and potential for controlled release. CNT polymer composites exhibit antimicrobial qualities, making them suitable for healthcare and food packaging. These materials can be used to develop antimicrobial coatings, wound dressings, and food packaging materials [108]. In today's industry, CNT/polymer composites are increasingly utilized in 3D printing technologies, that enhance mechanical and thermal properties. Enables to create of lightweight and high-strength components with complex geometries used for aerospace and automotive industries, where weight reduction and performance optimization are critical.

## 9 Conclusion

This comprehensive review paper underscores the increasing interest in the combination of CNTs with polymers to create advanced materials exhibiting enhanced performance characteristics. The primary focus of the research lies in incorporating CNTs into polymer matrices to improve mechanical, electrical, thermal, and physical properties, offering alternative solutions to materials with undesirable traits such as high density, low strength, and poor conductivity. Key conclusions include.

• The utilization of CNTs in polymer composites holds significant promise for enhancing mechanical, electrical, and thermal properties, resulting in lighter, stronger, more conductive, and durable materials.
• Polymers, when combined with CNTs, offer cost-effective, reproducible, corrosion-resistant, and easily manufacturable solutions, making them well-suited for various applications.
• The production process of CNT polymer composites requires optimization to achieve desired characteristics, considering factors like the type of polymer, intended application, and target properties.
• Ongoing research and development efforts focus on improving manufacturing processes for CNT polymer composites, aiming to enhance performance and tailor characteristics for diverse applications.
• The incorporation of CNTs enhances printability, augments specific material properties, or introduces novel ones in filament extrusion and AM processes.
• Applications of CNT polymer composite materials span across various industries, including transportation, automotive, aerospace, defense, sports, energy, and infrastructure.
• The available literature does not highlight any major gaps in knowledge, except for the need for further optimization of extrusion parameters to ensure consistent filament quality and the requirement for additional environmental impact assessments related to specific dispersion techniques.

In summary, the research on CNT polymer composites suggests significant potential for developing innovative materials with improved performance characteristics, especially in sectors such as transportation, automotive, aerospace, healthcare, and energy. Continued research and development efforts are essential to fully realize the potential of these materials and establish best practices for their production and application.

## Data availability statement

Data included in the article/supplementary material/referenced in the article.

## CRediT authorship contribution statement

**Ermias Wubete Fenta:** Writing – original draft, Visualization, Resources, Project administration, Methodology, Investigation, Formal analysis, Data curation, Conceptualization. **Berihun Abebaw Mebratie:** Writing – review & editing, Validation, Supervision.

## Declaration of competing interest

The authors declare that they have no known competing financial interests or personal relationships that could have appeared to influence the work reported in this paper.
